# The Prevention of Apoplexy
1This paper, although not a verbatim report, is substantially the address given by Professor Clifford Allbutt at the Meeting of the Bristol Medico-Chirurgical Society on January 11th, 1905.


**Published:** 1905-03

**Authors:** T. Clifford Allbutt

**Affiliations:** Regius Professor of Physic, University of Cambridge


The Bristol

Medico-Chirurgical Journal.

"*Scire est nescire, nisi id me

Scire alius sciret.*"

MARCH, 1905.

### THE PREVENTION OF APOPLEXY.[1]

BY

T. CLIFFORD ALLBUTT, M.D., F.R.S.,

*Regius Professor of Physic, University of Cambridge.*

---

FROM the time of Hippocrates physicians have aimed, by methods better and worse, at the forecast of disease. They have perceived that successful forecast is not only of prime utility in the particular case, but is the test by which they must be judged concerning their knowledge of the causes of disease, a knowledge in which must lie, in the long-run, our command of the means of cure. And if, leaving the particular instance, we turn our eyes towards the broader incidence of disease, we shall see that a knowledge of causes is the only way to what is far more than individual cure, namely prevention. On such con-

[1] This paper, although not a verbatim report, is substantially the address given by Professor Clifford Allbutt at the Meeting of the Bristol Medico-Chirurgical Society on January 11th, 1905.

siderations as these we may be content to be judged to-day. To the great Italians of the early renascence we owe far more than we are wont to acknowledge. To them we owe not indeed Harvey himself, but surely the spirit and the teaching which made Harvey what he was; and as in Harvey physiology began, so pathology had its chief source and inspiration in Morgagni.

Virchow has said that the key to Morgagni's reform was the substitution of the question, Where is disease? for What is disease?—the substitution of an inquiry into the place and order of the phenomena, instead of that which had ruled the Middle Ages, the inquiry into the essence of disease. Since Morgagni's day the revelations which have rewarded this change of attitude and method have been prodigious, and not in the direct results of anatomical search only. By the new method wide and deep changes have penetrated thence into the fields of clinical and therapeutical knowledge. In therapeutics, for instance, the distance between Morgagni and Wilks was as great as in morbid anatomy itself. The reform was sound, useful and progressive, almost above our appreciation. Yet, like all reforms, it has had its defect or partiality. To ask, as Virchow put it, Where is disease?—unless we give an infinite extension to the word "where"—is to convey too stationary a sense to the problem; to make it too static. Among the consequences of this limitation was a certain fatalism, both of pathology and of therapeutics; and this the more that, as in the vast majority of cases the necropsy does not take place till the disease has wrecked the organs affected, the mind is impressed by the destructive and inevitable aspect of the event, rather than by the processes, often very protracted and insidious, in which the event was generated. It is recognised on all hands that from this static attitude of observation prognosis and therapeutics suffered much loss; and during the last decennium the acuter observers of clinical phenomena have been engaged in encouraging a less fatalistic prognosis in diseases of the heart and kidneys, in tuberculosis, and in many other maladies. In diseases of the nervous system, as we should expect in the sphere of greatest complexity, one in which the causes are more profoundly withdrawn from direct observation, this fatalism still oppresses the physician. Where these diseases are seated is but too apparent; but by what processes they accumulate is as yet concealed from us. Now the ravages of disease are grievous enough without the despondencies of the *post-mortem* room. We shall gain heart, and the patient will gain hope, if we turn our eyes for a little while from this theatre to the clinical laboratory, in search of the genesis of disease; in an endeavour to detect the first small beginnings, which, unchecked, issue in such miscarriage. We shall not indeed go back to inquire, What is disease? but we shall not stop at the morbid anatomist's question, "Where is disease?"; we shall ask farther, How is disease?

Clinically, we have given up the catastrophic notion of disease; we have learned that its catastrophes are sudden only to him who is blind to their approaches. The springing of a mine is astonishing to its victims, but is no surprise to the sapper who laid it, who carried the clues to his tent, and at the just moment touched the button. The very name of apoplexy —in Latin, *sideratio*—signifies a stroke as if from the stars; the victim is, as it were, planet-stricken. And so it appeared to our fathers who gave it the name, and to many a generation after them; nay, so it appears still to the inexpert public. Yet nowadays the pathologist himself—possessed at first with fatalist submission, silent before the violent outbreak of blood into the delicate web of the brain, pondering in helpless dismay what man could have done in face of such a stroke—has begun to try to get behind the catastrophe. Now he is demonstrating to the bystander that granular kidneys, perhaps,—at any rate damaged arteries, and an abnormal heart, are precedent conditions. So that the event is surprising only to the unwarned. At this stage the eager mind questions farther and farther—how comes it that these arteries, these kidneys, this heart, are so changed? For all this is no swift infliction; it signifies modifications implying a long duration and gradual progress, modifications which again cannot have been without their insidious causes to take us farther back still; and so on. Thus, as in tuberculosis, we are laying aside the attitude of amazement or resignation, and are putting on that of the scout; if perchance we may detect the first approaches of the enemy, or even by timely diplomacy prevent him before war is declared. We are receding from the fatalism of the early pathological anatomists, and returning to that desire for more and more timely forecast which distinguished the schools of Cos and Cnidus; the forecast in which lie the proof of scientific knowledge and the means of prevention.

At the present time we are enthusiastic in the foreknowledge and prevention of tuberculosis; we are waylaying the epidemics in their courses; we are ardently pursuing the tracks of cancer; and as one by one we disarm them, we are gathering understanding and hope. It is my desire to-day to bring you to a like encouragement in respect of the apoplexy of cerebral hemorrhage.

That cases of "stroke" are not all the same kind, we have known for some time past; especially since the researches of Kirkes. On the cases in which healthy arteries are blocked by casual embolism, however, I have not now to speak; moreover, we will set aside all cases in which the effects of extrinsic poisonous or bacterio-toxic agents are concerned. We are to consider those in which disease of long-standing is found in the arteries about the seat of the hemorrhage. In a large number of these cases, however, we find no effusions of blood, or none in bulk, at any rate; the circulation of the brain is arrested, but by a silting up of the arteries rather than by rupture of them. Moreover, in these cases we find that the heart, abnormal as it may be, does not indicate present or previous hypertrophy; often indeed an atrophy. We find too that the arteries of these cases often present calcification of the middle coat, while the body at large is one in which senile change is far advanced, and probably not advanced prematurely—the patients do not run between sixty-five and seventy, but between seventy-five and eighty-five. In apoplexy by cerebral hemorrhage, the outbreak in the brain is no fault of this organ but wholly its misfortune. By apoplexy we lose day by day able citizens whose mental powers before the fatal seizure were intact both in vigour and quality. The pathological signs are those of some slow injury to the blood-vessels; but the heart is or has been hypertrophied, and the result of the conditions is rupture rather than occlusion.

Now what do we know, or what can we find out, concerning these awful visitations? For the last quarter of a century I have taught that in a large number of cases of sanguineous apoplexy the kidneys are not granular; and if in some of them they are fibrous, they do not partake of the nature of chronic Bright's disease. This I affirm on the condition of the secreting structures of the tubes, which dwindling or crushed as they may be here or there, present no foci, or traces of past foci, of degeneration or necrosis. Professor Osler has given his valuable judgment in favour of the proposition that a large number of cases of the kind we are contemplating are not attributable to chronic Bright's disease. Now my belief is that, if we can carry our analysis of causes far enough back, we shall reach a junction where we shall travel on a line of common approach to apoplexy with Bright's disease and to apoplexy without it; but for present convenience, and under the restriction of time, I must rule out the Brightian class. It is by the study in the first instance of the simpler case that we shall get back to the junction.

Now in a case of apoplexy what do we find in the damaged parts? Brain assumably healthy; heart hypertrophied; arteries spoiled: the phenomena lie then in the mechanism of the circulation. Thus, in accordance with our desire, we step back from the static point of view and enter upon the dynamic. We shall try to discover which of the variables in this function are altered? In a simple case the heart presents no primary changes, but changes altogether secondary; essentially it is not only healthy in tissue but has worked for a long time at high pressure, thus doing not less but more than its contract. Such changes as may be seen in it are compensatory, or, if morbid, evidently consequential. Then what about the arteries? These have undergone a change, call it atheroma, sclerosis—what you will, so long as we are agreed on signification—but, diseased as they are, they have not silted up, as in the cases we contemplated but to put aside, but have burst. Why have they burst? Because they have been submitted not only to the mean pressures of age but also to the augmenting mean pressures of a reluctant peripheral circulation. They have burst at last for the same reason that they have sustained gradual injury; namely by the accumulation of obscure stresses, which, if we might observe and measure them, we might avert and interpret. I put aversion before interpretation because happily in many conditions of life we can take up our guard before we know why we are the object of hostility, or even before we recognise our enemy. We do not know why in cases such as these the circulation is embarrassed: the cause of the reluctance in the periphery lies still beyond our ken. But, briefly, I may say that the cause must consist either in a narrowing of the calibres of the arteries or stream bed over a very extensive area, if not indeed universally, or in an increase of viscosity with excessive friction in the blood itself. I have been asked somewhat tartly how I demonstrate excess of viscosity, and in what it consists? My answer is, that I never said that the blood in these cases is more viscous, but that there exist the two alternatives only which I have cited —narrowing of the channels and increased friction within the fluid itself. To decide which is the cause, or, if both, the degree of each in the combination, I never pretended. But I admit that it is not easy for me to conceive a contraction of arteries in all or virtually all areas without compensatory dilatation in some of them. It has been suggested to me that in elderly persons the depressor property of the heart and vaso-dilatation may be stiffened or abolished. But a simple test will indicate that our vaso-dilator mechanism is not much abated. Let an elderly man enter a hot bath. For a short time at first he will find the radial artery contracting; let him continue however to observe, and in two or three minutes he will find the artery beginning to dilate, until it is largely distended; and a corresponding afflux of blood takes place to the surface. This is not dilatation of the splanchnic area, it is true; but if vaso-dilator mechanism does not rust up in one area, it probably does not in other areas.

It is alleged that in the elderly the arteries become refractory because of sclerosis, whereby their walls grow sluggish or stiff. This explanation, by the way, is inconsistent with that which attributes excessive arterial pressures to arterial contraction over large areas. And in any case to attribute high pressure to sclerosis, and to overlook the large class of cases in which arterial degeneration is manifested without rise of pressure is bewildering. Again, by some writers increase of arterial pressure is explained as a "hypertonus" of the arteries, a resuscitation surely of that older pathology which used to attribute disease to "hypertrophy of the heart"? It is conceivable, of course, that a morbid state of the vaso-motor centre, due to some persistent irritation in the spot, might keep up general and persistent vaso-motor contraction. Still this is not very probable, nor do I know that this is the view of those who discuss "hypertonus." Must we not assume for the present that hypertrophy in the arteries is produced by the same mechanism as in the heart, namely by persistently excessive pressures on their internal surfaces? In my opinion the vice lies not in a morbid tone of the vessels, but in excessive internal pressures such as obstruction at the periphery would set up. If, then, arterial spasm be also a factor in the hyperpiesis, it seems more consistent to attribute this to the same cause as that, whatever it may prove to be, which chokes the periphery. My observations are that in some cases of rising pressures without Bright's disease arterial spasm, whether primary or consequential, is manifestly present; but in others, perhaps the majority, it is not obvious. In some we have what I have called the "large, lax and leathery" artery; in others we find the "wiry" artery. The first kind may be regarded as "arterial tension," for in these cases the effects of tension are very manifest in the consequent tortuosity of the vessels; in the walls of wiry vessels this stretching effect, and indeed the sclerosis itself, is less apparent. Yet in my experience the wiry hyperpiesis is far more difficult to reduce.

However, to come to the matter of prevention; if, concerning the mechanism of persistent rise of mean arterial pressure, we are in the dark, happily there is less doubt as to the treatment of the condition. If the patient is to be saved from an apoplexy, it is only by long anticipation that the proclivity can be counteracted. It seems probable that a disposition to hyperpiesis runs in families; if so, in such families vigilance is imperative. But the tendency is too common to be regarded as one confined by any such hereditary limits. Even in children and youths it is by no means rare, though I have little information on the deferred consequences of hyperpiesis in such patients. Such information must be obtained from the family physician, who watches children from infancy to youth, from youth to maturity. This I can say, that in young people it may thicken the arteries plainly enough; but the thickening is probably of the muscular coat only, not of the intima, for it will disappear, as a hypertrophy of the heart disappears in persons who put aside causes of exceptional stress on this organ. The care of these juvenile cases, then, does not fall so near the group of potential apoplectics as to require our attention to-day. Still, I think a study of these precocious cases may throw light not only on an undescribed disorder of children, but also upon the causes of hyperpiesis in the elderly.

The aim of this discourse is to prevent apoplexy, which is a message to elderly persons. I have held against all comers for many years that arterio-sclerosis, as distinguished from the sclerotic decay of senile involution, is not the cause but the consequence of rising arterial pressures. In my view, then, prevention must lie first in the detection of a special tendency to a persistent mean rise. I need not say that occasional rises, even of morbid origin, are apt to occur in all persons, and to disappear before the vessels are permanently damaged. In others, however, the rise is persistent, often to very high degrees; yet if this tendency be detected in its earlier phases it can—in many instances, at any rate—be reduced and kept down; but the longer the story, the older the rearrangement of parts, the harder reduction becomes. I urge, then, that as a matter of routine every adult of the age of forty and upwards should have his blood pressures measured by the best instruments available, instruments which I have not now time to discuss. And I urge that this appreciation should be repeated every five years, say till the age of sixty, when, if there be no great increase—I say no great increase, for in almost all elderly persons there is some rise of mean pressure—the danger of apoplexy may be disregarded.

Of the principles of treatment of hyperpiesis we cannot be completely assured till the obscure points I have mentioned are cleared up. That there is any difference in treatment between the leathery and the wiry artery people I cannot say. So far I have not been able to discover more than that, as I have hinted, in the latter the perversion is far less submissive to deobstruent treatment, as generally understood, than in the former. Nor can I find any therapeutical divergence of practical value between burly, red-faced people and the spare and pallid. I am disposed to think, however, that pallor and wiry vessels are more frequent among the sedentary, and that the burly, red-faced people are of those who may over-eat and over-drink themselves, but take, on the other hand, much exercise in the fresh air. In the former there is a long history of relative excess in feeding; in the latter of positive excess. It cannot be too earnestly declared that nearly all men, and not a few women, take far more food than they need; and that the sedentary persons, such as scholars, lawyers, or merchants, although prompted by some nervous exhaustion they live more generously than cowboys, need very much less food than they habitually consume. If, then, in any person we find persistent rise of mean pressures, we shall revise his mode of life; advising regulated exercise, abstinence from alcohol—which, if not an initiator, is a potent ally of other influences—and a great reduction in intake of food. In these cases also, the regimen and the waters of certain spas—such as Harrogate, Carlsbad, or Marienbad—are invaluable.

The readiness of response in individuals is very various. In some, as I have said, reduction is attended with much difficulty; in others a couple of seasons at a bath, with punctilious restriction of diet and regular exercise, suffice to put the danger aside, at any rate for a time. In others, do what we may with regimen and medicines such as mercury and salines incessantly brought to bear, the rise, even if set back, comes up again and again. In such persons the ultimate result of apoplexy is pretty certain. It seems probable that the systematic blood-lettings of our forefathers, who were big feeders, was a rough-and-ready method of preventing morbid augmentations of blood pressures; and I am disposed to think that, practised with more discrimination, we might find in it a valuable remedy in the habit of body I have alluded to. I must honestly confess, however, that I have not had the moral courage to recommend it. Vaso-dilators are, as we should expect, disappointing. The high pressure is conservative, so that to reduce the pressure without removing or relieving the causes which import it is to set natural readjustments at naught. So long as the high pressures can persist the blood is driven through the periphery, and the patient may feel well enough; it is when the cardiac energy begins to slacken, and vaso-dilators are apt to slacken the heart also, that he suffers from the sense of exhaustion, the vertigo and the morning melancholy which vaso-dilators bring on factitiously. Notwithstanding, vaso-dilators may on occasion aid us at critical moments.[1]

I need not say that if a slight apoplexy occur, these measures must be undertaken with the more determination. Too often, unfortunately, we are not consulted until the enemy is upon us; still, even then, on the lines I have indicated, a return of the attack may be postponed with no little success.

In conclusion, let me urge upon you in all cases in which you are consulted by middle-aged persons, to note the blood pressures, and if possible to record them by means of one of the instruments which give us at any rate approximate estimates in this research. Not rarely, in consultation with physicians whose ability is above my praise, I note high blood pressures which they had not heeded, although they may freely admit the truth of the observation when their attention is drawn to it. Even the most capable of us are apt to see what we expect to see, and that only. Moreover the most erudite finger cannot always be trusted. It is my purpose, therefore, to invite you to take heed to the state of pressure in all middle-aged patients, and, if occasion occur, in persons who, not admitting any ill-health, may nevertheless be breeding an apoplexy unawares; a few years more neglect, and the event, unless anticipated by a fatal pneumonia, may be inevitable.

1 Dr. Oliver, of Harrogate, has had much experience in pressure reduction, and, I believe, relies to some extent on reduction by drugs.

